# Delayed Modeling Approach to Forecast the Periodic Behavior of SARS-2

**DOI:** 10.3389/fmolb.2020.585245

**Published:** 2021-04-01

**Authors:** Zhenhua Yu, Ayesha Sohail, Alessandro Nutini, Robia Arif

**Affiliations:** ^1^Institute of Systems Security and Control, College of Computer Science and Technology, Xi'an University of Science and Technology, Xi'an, China; ^2^Department of Mathematics, Comsats University Islamabad, Lahore, Pakistan; ^3^Centro Studi Attività Motorie, Biology and Biomechanics Department, Lucca, Italy

**Keywords:** SARS-CoV2, dynamical analysis, kinetic modeling, numerical simulations, monoclonal antibody, theoretical analysis

## Abstract

The ongoing threat of Coronavirus is alarming. The key players of this virus are modeled mathematically during this research. The transmission rates are hypothesized, with the aid of epidemiological concepts and recent findings. The model reported is extended, by taking into account the delayed dynamics. Time delay reflects the fact that the dynamic behavior of transmission of the disease, at time *t* depends not only on the state at time *t* but also on the state in some period τ before time *t*. The research presented in this manuscript will not only help in understanding the current threat of pandemic (SARS-2), but will also contribute in making precautionary measures and developing control strategies.

## 1. Introduction

In the field of biological sciences, the delayed processes takes place, not only at macro scale but also at micro scale. The computational framework for such problems can help in understanding the dynamics in a more cost effective manner (Yan, 2007; Fang et al., 2020; Sohail and Nutini, 2020).

Recently, the World Health Organization (WHO) has declared the novel corona virus (2019-nCoV) outbreak a Public Health Emergency of International Concern (PHEIC) It is named as severe acute respiratory syndrome corona virus 2 (SARS-CoV-2). The SARS-CoV-2 has been determined as the seventh member of the corona viruses infected humans (Zhu et al., 2020).

The antiviral immune response, also in the case of SARS-CoV2, behaves according to two characteristics: “lytic” and “non-lytic.” These characteristics emerge from the action of two fundamental elements of the immune system in the case of an anti-viral response: antibodies (non-lytic response) and cytotoxic T cells also called CTL—Cytotoxic Lymphocites (lytic response).

At the moment the epitopes that can be identified to analyze these responses, are not clear. There are strong homologies with the SARS-CoV virus (75.5%). Furthermore, there is a strong alteration of antigenicity compared to SARS-CoV2 even if important epitopes in the Spike protein have been identified through the analysis of the localization of the RDB sequences, which can therefore become specific targets for drugs and vaccines (Zheng and Song, 2020).

The non-lytic response occurs against adaptive humeral immunity through the production of antibodies by B cells whose action is to neutralize the virion through direct connection with the same; specific antibodies are important in the defense against viruses during the early stages of infection when the virus is still extra-cellular; neutralizing antibodies facing the virus bind to the capsid or viral pericapside proteins, preventing their adhesion to the cell surface and therefore entry into the cells. Opsonizing antibodies can potentate the elimination of viral particles by phagocytosis.

In the case of SARS-CoV2, virus-specific IgG reached 100% approximately 17–19 days after symptom onset, while the proportion of patients with positive virus-specific IgM reached a peak of 94.1% approximately 20–22 days after symptom onset while during the first 3 weeks after symptom onset, there was increase in virus-specific IgG and IgM antibody titters.

Three types of seroconversion are possible: synchronous seroconversion of IgG and IgM, IgM seroconversion earlier than that of IgG and IgM seroconversion later than that of IgG (Long et al., 2020); specific data on the production of IgG and IgM is crucial to allow the rapid identification of the infection (di Mauro Gabriella et al., 2020). The lytic response depends on the action of the CTL cells which, in order to take place efficiently, must result from a cooperation between *CD*4^+^ and *CD*8^+^ lymphocytes. *CD*8^+^ cells recognize endogenously synthesized viral antigens in association with MHC class I molecules on all cell types; *CD*4^+^ cells recognize viral antigens in association with MHC class II antigens and the complete differentiation of *CD*8^+^ cells requires the intervention of *CD*4^+^ T helper cells. The antiviral effect of CTL is due to the lysis of infected cells, the activation of endocellular enzymes (endonucleases) which cause the degradation of the viral genome and the secretion of cytokines with interferon activity.

CTL epitopes of SARS-CoV-2 have been predicted by several studies, which can be used effectively to understand the pathogenesis (Kumar et al., 2020).

The fundamental mechanism by which the advancement of the virus in an organism is contrasted is given by a balance between the two mechanisms (lytic and non lytic). During multiple stages of the infection, the initial coordinated action of the antibodies join the lytic activity of the CTL cells to eradicate the virus.

The current research is motivated by the possible lack of the “control” and “coordination” between the two types of immune response, such that, a strong CTL response induces a limited and insufficient antibody action (or the reverse); is interlinked with the strong decrease in viral load induced by one of the two types of response.

During this imbalanced process, the coordinative response of the *CD*4^+^ helper T cells also varies considerably: in SARS-CoV-2 infected patients, it has been reported that the analysis showed activation and reduction in *CD*4^+^ and *CD*8^+^ T cell counts (Li et al., 2020).

The purpose of this paper, therefore, is to quantify a possible mathematical model that investigates the possible non-coordination between lytic and non-lytic response and indicate a mathematical quantification of the best antiviral immune response in the case of SARS-CoV2.

## 2. Materials and the Methods

### 2.1. Basic Model of Virus Transmission

Consider the basic transmission model, with the coefficients, as listed in Table 1.

**Table 1:** Parameters description.

**Symbols**	**Description**	**Value**	**References**
ρ	Rate of generation of Susceptible host cells	110	Wodarz, 2005
*d*	The death rate of suspected host cells	0.01	Wodarz, 2005
μ	The replication rate of the virus	0.01	Wodarz, 2005
*a*	The death rate of infected cells	0.01	Assumed
*p*	The strength of the lytic component	1	Wodarz, 2005
κ	Rate of infected cells produced in Free virus	1	Assumed
*q*	The neutralization death rate by antibodies	1	Wodarz, 2005
ϕ	Decays rate in free virus	1	Assumed
*g*	Rate of Antibodies develop in response to free virus	20.5	Assumed
*h*	Rate of decays in Antibodies response	0.1	Wodarz, 2005
*c*	Rate of CTL response to viral antigen derived from infected cells	20.5	Assumed
*b*	Rate of decays in the absence of antigenic stimulation	0.1	Assumed

#### Model

(1) $$\begin{array}{l} \begin{array}{l} \frac{dU}{dt}=\rho -dU-\mu VU,\\ \frac{dW}{dt}=\mu VU-aW-pWC,\\ \frac{dV}{dt}=\kappa W-qVA-\phi V,\\ \frac{dA}{dt}=gVA-hA,\\ \frac{dC}{dt}=cWC-bC. \end{array} \end{array}$$

The description of compartments *U*(*t*), *W*(*t*), *V*(*t*), *A*(*t*), and *C*(*t*) are presented in Table 2. The basic concept of modeling was adapted from the work of Wodarz (2005).

**Table 2:** Compartments and their description.

**Symbols**	**Description**
*U*(*t*)	Susceptible host cells
*W*(*t*)	Infected cells
*V*(*t*)	Free virus
*A*(*t*)	Antibody response
*C*(*t*)	CTL response

#### 2.1.1. Positivity of Solution

For the model (1) transmission to be epidemiologically feasible, it is necessary to show that for all time every state variable is non-negative. Thus, the solutions of the model with non-negative initial value for all time *t* > 0 will remain non-negative.

**Theorem 2.1**. *Suppose that the model (1) consists of all feasible solutions with non-negative initial result then it remains non-negative for all time *t**.

**Proof:** Let the model (1) satisfy the initial non-negative solution, i.e.,

*U*(0) ≥ 0, *W*(0) ≥ 0, *V*(0) ≥ 0, *A*(0) ≥ 0 and *C*(0) ≥ 0.

It can be deduced from the model (1)

(2) $$\begin{array}{l} \frac{dU}{dt}=\rho -dU-\mu VU, \end{array}$$

as the solution of variable *U* can be computed by following result,

(3) $$\begin{array}{l} U=U\left(0\right)e^{G}+\int_{0}^{t}\pi e^{H\left(k\right)}d\left(k\right)\geq 0, \end{array}$$

where $G=-\int_{0}^{t}G\left(k\right)d\left(k\right)$ and $H\left(k\right)=-\int_{0}^{t}G\left(l\right)d\left(l\right)$. This gives non-negativity of *U*(0) ≥ 0 for all *t* ≥ 0. The non-negativity of the rest of the variables in the model (1) is given below.

(4) $$\begin{array}{l} \begin{array}{l} \frac{dW}{dt}=\mu VU-aW-pWC,\\ \frac{dV}{dt}=\kappa W-qVA-\phi V,\\ \frac{dA}{dt}=gVA-hA,\\ \frac{dC}{dt}=cWC-bC. \end{array} \end{array}$$

In term of matrix the above can be expressed as,

(5) $$\begin{array}{l} \frac{dF\left(t\right)}{dt}=H\left(t\right)+MF\left(t\right) \end{array}$$

where

(6) $$\begin{array}{l} F\left(t\right)=\left(\begin{array}{l} W\left(t\right)\\ V\left(t\right)\\ A\left(t\right)\\ C\left(t\right) \end{array}\right),H\left(t\right)=\left(\begin{array}{l} a\\ 0\\ 0\\ 0 \end{array}\right) \end{array}$$

and M matrix.

(7) $$\begin{array}{l} M=\left(\begin{array}{cccc} -a & \frac{\mu \rho }{d} & 0 & 0\\ \kappa & -\phi & 0 & 0\\ 0 & 0 & -h & 0\\ 0 & 0 & 0 & -b \end{array}\right). \end{array}$$

The matrix M is a Matzler matrix so by the result presented in Smith (1996) the model is monotone. The fact that $R_{+}^{4}$ is invariant with respect to stream of model (1).

#### 2.1.2. Qualitative Analysis

Stability analysis is an essential key to validate the models in the field of science and technology. The remarkable contributions by Tunc (2002); Tunç (2004, 2008, 2013) and other researchers in the cross references can not be denied, since their contribution help the researchers to deal with the highly nonlinear models.

Here we present the stability analysis of given mathematical model (1). The model (1) is locally asymptotically stable at uninfected and infected equilibrium points. For uninfected equilibrium, the model is locally stable, if the value of reproduction number ℝ_0_ < 1, whereas for infected equilibrium the model is stable if the value of the basic reproduction number ℝ_0_ > 1. Furthermore, we will investigate the model (1) is locally stable at uninfected and infected. The uninfected equilibrium point *E*^0^ = (*U*^0^, 0, 0, 0, 0) and infected equilibrium point E^*^ = (U^*^, W^*^, V^*^, A^*^, C^*^) of model (1) are constructed by following theorems.

**Theorem 2.2**. *The uninfected equilibrium point *E*^0^ of the model (1) is given by*

$$\begin{array}{l} E^{0}=\left(U^{0},0,0,0,0\right), \end{array}$$

where:

$$\begin{array}{l} U^{0}=\frac{\rho }{d}. \end{array}$$

**Proof:** By putting the equations of model (1) equal to zero, the dynamics is determined as follows:

$$\begin{array}{r} \begin{array}{r} \rho -dU-\mu VU=0,\\ \mu VU-aW-pWC=0,\\ \kappa W-qVA-\phi V=0,\\ gVA-hA=0,\\ cWC-bC=0, \end{array} \end{array}$$

and after further algebraic manipulation, we got

(8) $$\begin{array}{l} U^{0}=\frac{\rho }{d}, \end{array}$$

which completes the proof.

**Theorem 2.3**. The model (1) admits a unique infected equilibrium E^*^ = (U^*^, W^*^, V^*^, A^*^, C^*^) if and only if ℝ_0_ > 1.

**Proof:** The infected equilibrium point is given as:

(9) $$\begin{array}{l} \;U^{*}=\frac{g\rho }{dg+\mu h},\\ W^{*}=\frac{b}{c},\\ \;V^{*}=\frac{h}{g},\\ \;A^{*}=\frac{bg\kappa -ch\phi }{chq},\\ \;C^{*}=\frac{-abdg-ab\mu h+\mu ch\rho }{bp\left(dg+\mu h\right)}. \end{array}$$

#### 2.1.3. Reproduction Number ℝ_0_ and Stability Analysis

The basic reproduction number ℝ_0_ is formulated by evaluating infection matrix **F** and transmission matrix **V** constructed from Jacobian matrix *J* of model (1). The reproduction number ℝ_0_ is the spectral radius of matrix **FV**^−1^. The Jacobian matrix for *J*_0_ for uninfected equilibrium point is

(10) $$\begin{array}{l} J_{0}=\left(\begin{array}{ccccc} -d & 0 & -\frac{\mu \rho }{d} & 0 & 0\\ 0 & -a & \frac{\mu \rho }{d} & 0 & 0\\ 0 & \kappa & -\phi & 0 & 0\\ 0 & 0 & 0 & -h & 0\\ 0 & 0 & 0 & 0 & -b \end{array}\right) \end{array}$$

The infection matrix *F* and rest of transmission matrix *V* are give as follow:

(11) $$\begin{array}{l} \text{V}=\left(\begin{array}{ccccc} d & 0 & 0 & 0 & 0\\ 0 & a & 0 & 0 & 0\\ 0 & -\kappa & \phi & 0 & 0\\ 0 & 0 & 0 & h & 0\\ 0 & 0 & 0 & 0 & b \end{array}\right), \end{array}$$

(12) $$\begin{array}{l} F=\left(\begin{array}{ccccc} 0 & 0 & -\frac{\mu \rho }{d} & 0 & 0\\ 0 & 0 & \frac{\mu \rho }{d} & 0 & 0\\ 0 & 0 & 0 & 0 & 0\\ 0 & 0 & 0 & 0 & 0\\ 0 & 0 & 0 & 0 & 0 \end{array}\right). \end{array}$$

The matrix *K* = **FV**^−1^ is constructed as follows:

(13) $$\begin{array}{l} K=\left(\begin{array}{ccccc} 0 & -\frac{\mu \kappa \rho }{ad\phi } & -\frac{\mu \rho }{d\phi } & 0 & 0\\ 0 & \frac{\mu \kappa \rho }{ad\phi } & \frac{\mu \rho }{d\phi } & 0 & 0\\ 0 & 0 & 0 & 0 & 0\\ 0 & 0 & 0 & 0 & 0\\ 0 & 0 & 0 & 0 & 0 \end{array}\right). \end{array}$$

The spectral radius of matrix *K* = **FV**^−1^ give reproductive number of model (1) is

(14) $$\begin{array}{l} {\mathbb{R}}_{0}=\frac{\mu \kappa \rho }{ad\phi }. \end{array}$$

The stability analysis of system (1) is presented in following Theorem 2.4.

**Theorem 2.4**. *Uninfected equilibrium point *E*_0_, will be locally asymptotically stable if* ℝ_0_ < 1 *and otherwise it will be unstable.*

**Proof:** The Jacobian matrix *J*(*E*_0_) of model (1) for uninfected equilibrium point is formulated as follows:

(15) $$\begin{array}{l} J\left(E_{0}\right)=\left(\begin{array}{ccccc} -d & 0 & -\frac{\mu \rho }{d} & 0 & 0\\ 0 & -a & \frac{\mu \rho }{d} & 0 & 0\\ 0 & \kappa & -\phi & 0 & 0\\ 0 & 0 & 0 & -h & 0\\ 0 & 0 & 0 & 0 & -b \end{array}\right). \end{array}$$

The eigenvalues of Jacobian matrix *J*(*E*_0_) is evaluated by characteristic equation that is *det*(*J*(*E*_0_) − *Iλ*_*i*_) = 0, for *i* = 1 : 5. The eigenvalues are given as follows:

λ_1_ = −*b*, λ_2_ = −*d*, λ_3_ = −*h*, $\lambda_{4}=-\frac{\left(\sqrt{d}\right)\left(a+\phi \right)+\sqrt{d{\left(a-\phi \right)}^{2}+4\mu \kappa \rho }}{2\sqrt{d}}$ and $\lambda_{5}=-\frac{\sqrt{d}\left(a+\phi \right)-\sqrt{d{\left(a-\phi \right)}^{2}+4\mu \kappa \rho }}{2\sqrt{d}}$.

Thus, all eigenvalues λ_*i*_ are strictly negative for *i* = 1 : 5. Hence, model (1) is locally asymptotically stable.

** Lemma 2.5**. *If* *R*_0_ > 1, *the infected equilibrium point of system is locally asymptotically stable, otherwise it is unstable.*

**Proof:** The results can by obtained by the same procedure of (2.4).

#### 2.1.4. Sensitivity Analysis

The sensitivity of basis reproductive

(16) $$\begin{array}{l} {\mathbb{R}}_{0}=\frac{\mu \kappa \rho }{ad\phi } \end{array}$$

is analyze with respect to each parameters is follows:

(17) $$\begin{array}{l} \begin{array}{l} \frac{\partial {\mathbb{R}}_{0}}{\partial \mu }=\frac{\kappa \rho }{ad\phi }>0,\\ \frac{\partial {\mathbb{R}}_{0}}{\partial \kappa }=\frac{\mu \rho }{ad\phi }>0,\\ \frac{\partial {\mathbb{R}}_{0}}{\partial \rho }=\frac{\mu \kappa }{ad\phi }>0,\\ \frac{\partial {\mathbb{R}}_{0}}{\partial a}=-\frac{\mu \kappa \rho }{a^{2}d\phi }<0,\\ \frac{\partial {\mathbb{R}}_{0}}{\partial d}=-\frac{\mu \kappa \rho }{ad^{2}\phi }<0,\\ \frac{\partial {\mathbb{R}}_{0}}{\partial \phi }=-\frac{\mu \kappa \rho }{ad\phi^{2}}<0. \end{array} \end{array}$$

It is clear that reproductive number is directly proportional to the replication rate of the virus, rate of infected cells produced in free virus and rate of generation of susceptible host cells. Decrease with the death rate of infected cells, decays rate in free virus and the death rate of suspected host cells.

The basic reproductive number is ${\mathbb{R}}_{0}=\frac{\mu \kappa \rho }{ad\phi }>1$ in case of infection. In the absence of an immune responses the model (1) converges to the following equilibrium points *E*^(0)^ = (*U*^(0)^, *W*^(0)^, *V*^(0)^, *A*^(0)^, *C*^(0)^) which is defined as

(18) $$\begin{array}{l} U^{\left(0\right)}=\left(\frac{\rho }{d},0,0,0,0\right). \end{array}$$

We assumed that the immune responses is present. this desires following conditions: *cW*^(0)^ > *b* and *gV*^(0)^ > *h*. In this case following can be observed.

1. The anti body response can't established and the CTL response develops. Because the CTL response is strong and decrease virus load to levels which are vary low to stimulate antibody response. It has following equilibrium points,
  *E*^(1)^ = (*U*^(1)^, *W*^(1)^, *V*^(1)^, *A*^(1)^, *C*^(1)^) where,
  (19) $$\begin{array}{l} \begin{array}{l} U^{\left(1\right)}=\frac{\text{c}\phi \rho }{b\kappa \mu +cd\phi },\\ W^{\left(1\right)}=\frac{b}{c},\\ V^{\left(1\right)}=\frac{b\kappa }{c\phi },\\ A^{\left(1\right)}=0,\\ C^{\left(1\right)}=\frac{-ab\kappa \mu -acd\phi +c\kappa \mu \rho }{p\left(b\kappa \mu +cd\phi \right)}. \end{array} \end{array}$$
  This is obtained if $\frac{bg\kappa }{c\phi }<h$ and $\frac{ch\rho \mu }{a\left(dg+h\mu \right)}>b$.
2. The sustained CTL response zero and antibody response develop, because the antibody response is strong relative to CTL response and decrease virus load to levels which are vary low to stimulate CTL. It has following equilibrium points.
  $$\begin{array}{l} E^{\left(2\right)}=\left(U^{\left(2\right)},W^{\left(2\right)},V^{\left(2\right)},A^{\left(2\right)},C^{\left(2\right)}\right), \end{array}$$
  which is defined as follows:
  (20) $$\begin{array}{l} \begin{array}{l} \;U^{\left(2\right)}=\frac{g\rho }{dg+h\mu },\\ W^{\left(2\right)}=\frac{h\mu \rho }{a\left(dg+h\mu \right)},\\ \;V^{\left(2\right)}=\frac{h}{g},\\ A^{\left(2\right)}=\frac{-adg\phi -ah\mu \phi +g\kappa \mu \rho }{aq\left(dg+h\mu \right)},\\ C^{\left(2\right)}=0. \end{array} \end{array}$$
  This is obtained if $\frac{bgk}{c\phi }>h$ and $\frac{ch\rho \mu }{a\left(dg+h\mu \right)}<b$.
3. CTL and anti body response develops. This equilibrium points are as follows:
  (21) $$\begin{array}{l} E^{\left(3\right)}=\left(U^{\left(3\right)},W^{\left(3\right)},V^{\left(3\right)},A^{\left(3\right)},C^{\left(3\right)}\right) \end{array}$$
  where,
  (22) $$\begin{array}{l} \begin{array}{l} \;U^{\left(3\right)}=\frac{g\rho }{dg+h\mu },\\ W^{\left(3\right)}=\frac{b}{c},\\ \;V^{\left(3\right)}=\frac{h}{g},\\ \;A^{\left(3\right)}=\frac{bg\kappa -ch\phi }{chq},\\ \;C^{\left(3\right)}=\frac{ch\mu \rho -abdg-abh\mu }{bp\left(dg+h\mu \right)}. \end{array} \end{array}$$
  This is obtained if $\frac{bgk}{c\phi }>h$ and $\frac{ch\rho \mu }{a\left(dg+h\mu \right)}>b$.

### 2.2. Modified Modeling Approach

(23) $$\begin{array}{l} \begin{array}{l} \frac{dU}{dt}=\rho -dU-\mu \frac{VU}{1+\eta U},\\ \frac{dW}{dt}=\mu \frac{VU}{1+\eta U}-aW-pWC,\\ \frac{dV}{dt}=\kappa W-qVA-\phi V,\\ \frac{dA}{dt}=gVA-hA,\\ \frac{dC}{dt}=cWC-bC, \end{array} \end{array}$$

where the description of compartments *U*(*t*), *W*(*t*), *V*(*t*), *A*(*t*), and *C*(*t*) are presented in Table 2.

#### 2.2.1. Positivity of Solution

For the model (23) transmission to be epidemiologically feasible, it is necessary to show that for all time every state variable is non-negative. Thus, the solutions of the model with non-negative initial value for all time *t* > 0 will remain non-negative.

**Theorem 2.6**. *Suppose that the model(23) consists of all feasible solutions with non-negative initial result then it remains non-negative for all time *t*.*

**Proof:** Let the model (23) satisfy that the initial non-negative solution, i.e.,

*U*(0) ≥ 0, *W*(0) ≥ 0, *V*(0) ≥ 0, *A*(0) ≥ 0, and *C*(0) ≥ 0.

It can be deduced from model (23) that is,

(24) $$\begin{array}{l} \frac{dU}{dt}=\rho -dU-\mu \frac{VU}{1+\eta U}. \end{array}$$

As the solution of variable *U* can be computed by following result,

(25) $$\begin{array}{l} U=U\left(0\right)e^{G}+\int_{0}^{t}\pi e^{H\left(k\right)}d\left(k\right)\geq 0 \end{array}$$

where $G=-\int_{0}^{t}G\left(k\right)d\left(k\right)$ and $H\left(k\right)=-\int_{0}^{t}G\left(l\right)d\left(l\right)$. This gives non-negativity of *U*(0) ≥ 0 for all *t* ≥ 0. The non-negativity of rest variables in the model (23) is given as follows,

(26) $$\begin{array}{l} \begin{array}{l} \frac{dW}{dt}=\mu \frac{VU}{1+\eta U}-aW-pWC,\\ \frac{dV}{dt}=\kappa W-qVA-\phi V,\\ \frac{dA}{dt}=gVA-hA,\\ \frac{dC}{dt}=cWC-bC. \end{array} \end{array}$$

In term of matrix the above can be expressed as,

(27) $$\begin{array}{l} \frac{dF\left(t\right)}{dt}=H\left(t\right)+MF\left(t\right) \end{array}$$

where *F*(*t*) and *H*(*t*) are as follows:

(28) $$\begin{array}{l} F\left(t\right)=\left(\begin{array}{c} W\left(t\right)\\ V\left(t\right)\\ A\left(t\right)\\ C\left(t\right) \end{array}\right),H\left(t\right)=\left(\begin{array}{c} a\\ 0\\ 0\\ 0 \end{array}\right) \end{array}$$

and we have matrix M

(29) $$\begin{array}{l} M=\left(\begin{array}{cccc} -a & \frac{\mu \rho }{d\left(\frac{\eta \rho }{d}+1\right)} & 0 & 0\\ \kappa & -\phi & 0 & 0\\ 0 & 0 & -h & 0\\ 0 & 0 & 0 & -b \end{array}\right). \end{array}$$

The matrix M is a Matzler matrix so by the result presented in Smith (1996) the model is monotone. the fact that $R_{+}^{4}$ is invariant with respect to stream of model (23).

#### 2.2.2. Qualitative Analysis

Here we present the stability analysis of (23). The model (23) is locally asymptotically stable at uninfected and infected equilibrium points. We investigate the model (23) is locally stable at uninfected and infected. The uninfected equilibrium point *E*^0^ = (*U*^0^, 0, 0, 0, 0) and infected equilibrium point E^*^ = (U^*^, W^*^, V^*^, A^*^, C^*^) of model (23) are constructed by following theorems.

**Theorem 2.7**. *The uninfected equilibrium point* *E*^0^ *of the model (23) is given by*

$$\begin{array}{l} E^{0}=\left(U^{0},0,0,0,0\right), \end{array}$$

where:

$$\begin{array}{l} U^{0}=\frac{\rho }{d}. \end{array}$$

**Proof:** By putting the equations in (23) equal to zero, the dynamics is determined as follows,

$$\begin{array}{r} \begin{array}{r} \rho -dU-\mu \frac{VU}{1+\eta U}=0,\\ \mu \frac{VU}{1+\eta U}-aW-pWC=0,\\ \kappa W-qVA-\phi V=0\\ gVA-hA=0,\\ cWC-bC=0. \end{array} \end{array}$$

By solving these equations, we got

(30) $$\begin{array}{l} U^{0}=\frac{\rho }{d}. \end{array}$$

This completes the proof.

**Theorem 2.8**. *The model (23) admits a unique infected equilibrium* E^*^ = (U^*^, W^*^, V^*^, A^*^, C^*^) *if and only if* ℝ_0_ > 1.

**Proof:** Calculating the infected equilibrium point, we obtain

(31) $$\begin{array}{l} \begin{array}{l} \;U^{*}=\frac{-\sqrt{4d\eta g^{2}\rho +{\left(dg-\eta g\rho +h\mu \right)}^{2}}}{2dg\eta }\\ \;+\frac{\eta g\rho -dg-h\mu }{2dg\eta },\\ W^{*}=\frac{b}{c},\\ V^{*}=\frac{h}{g},\\ A^{*}=\frac{bg\kappa -ch\phi }{chq},\\ C^{*}=\frac{-ab-cdU^{*}+c\rho }{bp}. \end{array} \end{array}$$

#### 2.2.3. Reproduction Number ℝ_0_ and Stability Analysis

The basic reproduction number ℝ_0_ is formulated by evaluating infection matrix **F** and transmission matrix **V** constructed from Jacobian matrix *J* of system (23). The reproduction number ℝ_0_ is the spectral radius of matrix **FV**^−1^. The Jacobian matrix for *J*_0_ for uninfected equilibrium point is,

(32) $$\begin{array}{l} J_{0}=\left(\begin{array}{ccccc} 0 & 0 & -\frac{\mu \rho }{d\left(\frac{\eta \rho }{d}+1\right)} & 0 & 0\\ 0 & -a & \frac{\mu \rho }{d\left(\frac{\eta \rho }{d}+1\right)} & 0 & 0\\ 0 & \kappa & -\phi & 0 & 0\\ 0 & 0 & 0 & -h & 0\\ 0 & 0 & 0 & 0 & -b \end{array}\right). \end{array}$$

The infection and transmission matrices, *F* & *V* are give as follow:

(33) $$\begin{array}{l} \text{V}=\left(\begin{array}{ccccc} 0 & 0 & \frac{\mu \rho }{d\left(\frac{\eta \rho }{d}+1\right)} & 0 & 0\\ 0 & a & -\frac{\mu \rho }{d\left(\frac{\eta \rho }{d}+1\right)} & 0 & 0\\ 0 & -\kappa & \phi & 0 & 0\\ 0 & 0 & 0 & h & 0\\ 0 & 0 & 0 & 0 & 0 \end{array}\right), \end{array}$$

(34) $$\begin{array}{l} F=\left(\begin{array}{cccc} 0 & -\frac{\mu \rho }{d\left(\frac{\eta \rho }{d}+1\right)} & 0 & 0\\ 0 & \frac{\mu \rho }{d\left(\frac{\eta \rho }{d}+1\right)} & 0 & 0\\ 0 & 0 & 0 & 0\\ 0 & 0 & 0 & 0 \end{array}\right). \end{array}$$

The matrix *K* = **FV**^−1^ is constructed as follows:

(35) $$\begin{array}{l} K=\left(\begin{array}{cccc} -\frac{\kappa \mu \rho }{ad\left(\frac{\eta \rho }{d}+1\right)\phi } & -\frac{\mu \rho }{d\left(\frac{\eta \rho }{d}+1\right)\phi } & 0 & 0\\ \frac{\kappa \mu \rho }{ad\left(\frac{\eta \rho }{d}+1\right)\phi } & \frac{\mu \rho }{d\left(\frac{\eta \rho }{d}+1\right)\phi } & 0 & 0\\ 0 & 0 & 0 & 0\\ 0 & 0 & 0 & 0 \end{array}\right). \end{array}$$

The spectral radius of matrix *K* = **FV**^−1^ finally provides the reproductive number for given model (23):

(36) $$\begin{array}{l} {\mathbb{R}}_{0}=\frac{\mu \rho \left(a-\kappa \right)}{a\phi \left(d+\eta \rho \right)}. \end{array}$$

The stability analysis of system (23) is presented by follows theorem.

**Theorem 2.9**. *For uninfected equilibrium point* *E*_0_ *if the real part of eigenvalues of Jacobian matrix *J* of the system (23) are strictly negative, then the system (23) is locally stable otherwise it is unstable*.

**Proof:** The Jacobian matrix of model (23) for uninfected equilibrium point *J*(*E*_0_) is formulated as follows:

(37) $$\begin{array}{l} J\left(E_{0}\right)=\left(\begin{array}{ccccc} 0 & 0 & -\frac{\mu \rho }{d\left(\frac{\eta \rho }{d}+1\right)} & 0 & 0\\ 0 & -a & \frac{\mu \rho }{d\left(\frac{\eta \rho }{d}+1\right)} & 0 & 0\\ 0 & \kappa & -\phi & 0 & 0\\ 0 & 0 & 0 & -h & 0\\ 0 & 0 & 0 & 0 & -b \end{array}\right). \end{array}$$

The eigenvalues of Jacobian matrix *J*(*E*_0_) is evaluated by characteristic equation *det*(*J*(*E*_0_) − *Iλ*_*i*_) = 0, for *i* = 1 : 5. The eigenvalues are given as follows:

λ_1_ = 0, λ_2_ = −*b*, λ_3_ = −*h*,

$\lambda_{4}=\frac{1}{2}\left(\sqrt{{\left(a-\phi \right)}^{2}+\frac{4\kappa \mu \rho }{d+\eta \rho }}+a+\phi \right)$

and

$\lambda_{5}=\frac{1}{2}\left(-\sqrt{{\left(a-\phi \right)}^{2}+\frac{4\kappa \mu \rho }{d+\eta \rho }}+a+\phi \right)$.

Since the all eigenvalues λ_*i*_, satisfy the criteria (where as *i* = 1 : 5). Hence, the model (23) is locally stable.

#### 2.2.4. Sensitivity Analysis

The sensitivity of basis reproductive

(38) $$\begin{array}{l} {\mathbb{R}}_{0}=\frac{\mu \rho \left(a-\kappa \right)}{a\phi \left(d+\eta \rho \right)} \end{array}$$

is analyze with respect to each parameters is as follows:

(39) $$\begin{array}{l} \begin{array}{l} \frac{\partial {\mathbb{R}}_{0}}{\partial a}=\frac{\kappa \mu \rho }{a^{2}\phi \left(d+\eta \rho \right)}>0,\\ \frac{\partial {\mathbb{R}}_{0}}{\partial \mu }=\frac{a\rho -\kappa \rho }{ad\phi +a\eta \rho \phi }>0, \end{array}\\ \begin{array}{l} \frac{\partial {\mathbb{R}}_{0}}{\partial \rho }=\frac{d\mu \left(a-\kappa \right)}{a\phi {\left(d+\eta \rho \right)}^{2}}>0,\\ \frac{\partial {\mathbb{R}}_{0}}{\partial \kappa }=-\frac{\mu \rho }{ad\phi +a\eta \rho \phi }<0\\ \frac{\partial {\mathbb{R}}_{0}}{\partial d}=-\frac{\mu \rho \left(a-\kappa \right)}{a\phi {\left(d+\eta \rho \right)}^{2}}<0,\\ \frac{\partial {\mathbb{R}}_{0}}{\partial \phi }=-\frac{\mu \rho \left(a-\kappa \right)}{a\phi^{2}\left(d+\eta \rho \right)}<0,\\ \frac{\partial {\mathbb{R}}_{0}}{\partial \eta }=-\frac{\mu \rho^{2}\left(a-\kappa \right)}{a\phi {\left(d+\eta \rho \right)}^{2}}<0. \end{array} \end{array}$$

Therefore, the reproductive number ℝ_0_ is directly proportional to *a*, μ, and ρ and inversely proportional to κ, *d*, ϕ and η.

The basic reproductive number is ${\mathbb{R}}_{0}=\frac{\mu \kappa \rho }{ad\phi }>1$ in case of infection.

In the absence of an immune responses the model (23) converges to the following equilibrium points: *E*^(0)^ = (*U*^(0)^, *W*^(0)^, *V*^(0)^, *A*^(0)^, *C*^(0)^) which is defined as $U^{\left(0\right)}=\frac{\rho }{d},W^{\left(0\right)}=0,V^{\left(0\right)}=0,A^{\left(0\right)}=0,C^{\left(0\right)}=0$. We assumed that the immune responses is present. This desires following conditions: *cW*^(0)^ > *b* and *gV*^(0)^ > *h*.

In this case, the following can be observed:

1. The anti body response can not be established and the CTL response develops.
  Because of strong CTL response and low virus load to levels, it has the following equilibrium points:
  (40) $$\begin{array}{l} \begin{array}{l} E^{\left(1\right)}=\left(U^{\left(1\right)},W^{\left(1\right)},V^{\left(1\right)}A^{\left(1\right)},C^{\left(1\right)}\right),\\ E^{\left(1\right)}=\left(X,\frac{b}{c},\frac{b\kappa }{c\phi },0,Y\right), \end{array} \end{array}$$
  where:
  (41) $$\begin{array}{l} \;U^{\left(1\right)}=\frac{-b\kappa \mu -c\phi \left(d-\eta \rho \right)}{2cd\eta \phi },\\ \;-\frac{\sqrt{{\left(b\kappa \mu +c\phi \left(d-\eta \rho \right)\right)}^{2}+4c^{2}d\eta \rho \phi^{2}}}{2cd\eta \phi },\\ W^{\left(1\right)}=\frac{b}{c},\\ \;V^{\left(1\right)}=\frac{b\kappa }{c\phi },\\ A^{\left(1\right)}=0,\\ C^{\left(1\right)}=\frac{-ab-cdU^{\left(1\right)}+c\rho }{bp}. \end{array}$$
  This equilibrium points is obtain if it follows the following condition:
  $\frac{bg\kappa }{c\phi }<h$ and $\frac{c\sqrt{4d\eta g^{2}\rho +{\left(-dg+\eta g\rho -h\mu \right)}^{2}}+cdg+c\eta g\rho +ch\mu }{2ag\eta }>b$.
2. For null sustained CTL response, the antibody response develops. Because of strong antibody response to relative CTL response and low virus load to levels which is too small for stimulate CTL. It has the following equilibrium points:
  (42) $$\begin{array}{l} E^{\left(2\right)}=\left(U^{\left(2\right)},W^{\left(2\right)},V^{\left(2\right)},A^{\left(2\right)},C^{\left(2\right)}\right), \end{array}$$
  which is defined as follows:
  $$\begin{array}{l} \begin{array}{l} \;U^{\left(2\right)}=\frac{-dg+\eta g\rho -h\mu }{2dg\eta },\\ \;-\frac{\sqrt{4d\eta g^{2}\rho +{\left(dg-\eta g\rho +h\mu \right)}^{2}}}{2dg\eta },\\ W^{\left(2\right)}=\frac{g\rho }{a}-U^{\left(2\right)}, \end{array} \end{array}$$
  (43) $$\begin{array}{l} \begin{array}{l} V^{\left(2\right)}=\frac{h}{g},\\ A^{\left(2\right)}=\frac{g\kappa \left(\rho -dU^{\left(2\right)}\right)-ah\phi }{ahq},\\ C^{\left(2\right)}=0. \end{array} \end{array}$$
  The equilibrium point satisfies the following criteria: $\frac{bg\kappa }{c\phi }>h$ and $\frac{c\sqrt{4d\eta g^{2}\rho +{\left(-dg+\eta g\rho -h\mu \right)}^{2}}+cdg+c\eta g\rho +ch\mu }{2ag\eta }<b$.
3. CTL and anti body response develops. The equilibrium point is given as:
  (44) $$\begin{array}{l} E^{*}=\left(U^{*},W^{*},V^{*},A^{*},C^{*}\right) \end{array}$$
  where:
  (45) $$\begin{array}{l} \;U^{*}=\frac{-\sqrt{4d\eta g^{2}\rho +{\left(dg-\eta g\rho +h\mu \right)}^{2}}}{2dg\eta },\\ \;+\frac{\eta g\rho -dg-h\mu }{2dg\eta },\\ \;W^{*}=\frac{b}{c},\\ \;V^{*}=\frac{h}{g},\\ \;A^{*}=\frac{bg\kappa -ch\phi }{chq},\\ \;C^{*}=\frac{-ab-cdU^{*}+c\rho }{bp}, \end{array}$$
  under the criteria: $\frac{bg\kappa }{c\phi }>h$ and $\frac{c\sqrt{4d\eta g^{2}\rho +{\left(-dg+\eta g\rho -h\mu \right)}^{2}}+cdg+c\eta g\rho +ch\mu }{2ag\eta }>b$.

### 2.3. Delayed Model

Different biological models with delayed transmission are available in the literature (Maiti et al., 2008; Lv and Yuan, 2009; Kuniya and Nakata, 2012). The schematic 2 presents the transmission of the virus from one compartment to the other. The delay takes place after the interaction of virus with the target cells at τ_1_, it is further assumed that at delay τ_2_, the infected cells and the CTLs interact and the antibodies and the virus interact.

(46) $$\begin{array}{l} \begin{array}{l} \frac{dU\left(t\right)}{dt}=\rho -dU\left(t\right)-\mu \frac{V\left(t\right)U\left(t\right)}{1+\eta U\left(t\right)},\\ \frac{dW\left(t\right)}{dt}=\frac{\mu V\left(t-\tau_{1}\right)U\left(t-\tau_{1}\right)}{1+\eta U\left(t-\tau_{1}\right)}-aW\left(t\right),\\ \;-pW\left(t\right)C\left(t\right),\\ \frac{dV\left(t\right)}{dt}=\kappa W\left(t\right)-qV\left(t\right)A\left(t\right)-\phi V\left(t\right),\\ \frac{dA\left(t\right)}{dt}=gV\left(t-\tau_{2}\right)A\left(t-\tau_{2}\right)-hA\left(t\right),\\ \frac{dC\left(t\right)}{dt}=cW\left(t-\tau_{2}\right)C\left(t-\tau_{2}\right)-bC\left(t\right). \end{array} \end{array}$$

#### 2.3.1. Local Stability for Delay Differential Equations

In ODE's, the local stability of consistent condition relies upon the area of underlying foundations of characteristic function, that are polynomial in shape. The unfaltering condition is steady if the majority of the roots having −ve real part. The outstanding Routh-Hurwitz criteria provide exact situation for arbitrary polynomials. For DDE's, nearby stability is likewise controlled by the area of trademark work, yet for this situation, this capacity appears as an alleged quasi polynomial, that is supernatural. Hence, there are vastly numerous roots. Moreover, the Routh-Hurwitz criteria are not pertinent. Numerous methodologies decide the stability of steady states delay equations.

#### 2.3.2. Domain Subdivision

D-subdivision or Domain subdivision, utilizes basic details about the actions of the roots of characteristic functions, as a parameter modifies to split parameter space into parts where the no. of roots with +ve real parts is constant. The roots' position depends consistently on the models' parameter and when the parameters are altered, another root rises if imaginary root exists for a set of parameters.

Now we subdivide the parametric domain by hypersurfaces comprising of parameter routines for which at least one simply imaginary roots exist. When the districts are limited by these hypersurfaces, the quantity of roots with +*ve* real part is constant. Obviously, the locales where the number is zero and their supplements are more interesting. This technique is especially simple to picture when the framework in question relies upon two parameters, so the area is ℝ^2^, and, the hypersurfaces are curve.

## 3. Results and Discussion

During this research, we have used the Matlab^*TM*^ delay differential equations bifurcation analysis tools. For the validity of the computational model, extensive numerical experiments were conducted using simulink toolbox.

### 3.1. Analysis of SARS-CoV2 and Antibodies Interaction

With the aid of mathematical model we have concluded the following:

1. The analysis of lytic vs. non lytic immune response plays an important role in infection control.
2. The Hill function is important in kinetic modeling and the Hill coefficient is important parameter to forecast a complete cycle of infection.
3. The analytic approach and numerical Matcont bifurcation analysis proved to be efficient in parametric approximation for such complex dynamics.

**Figure 1:**
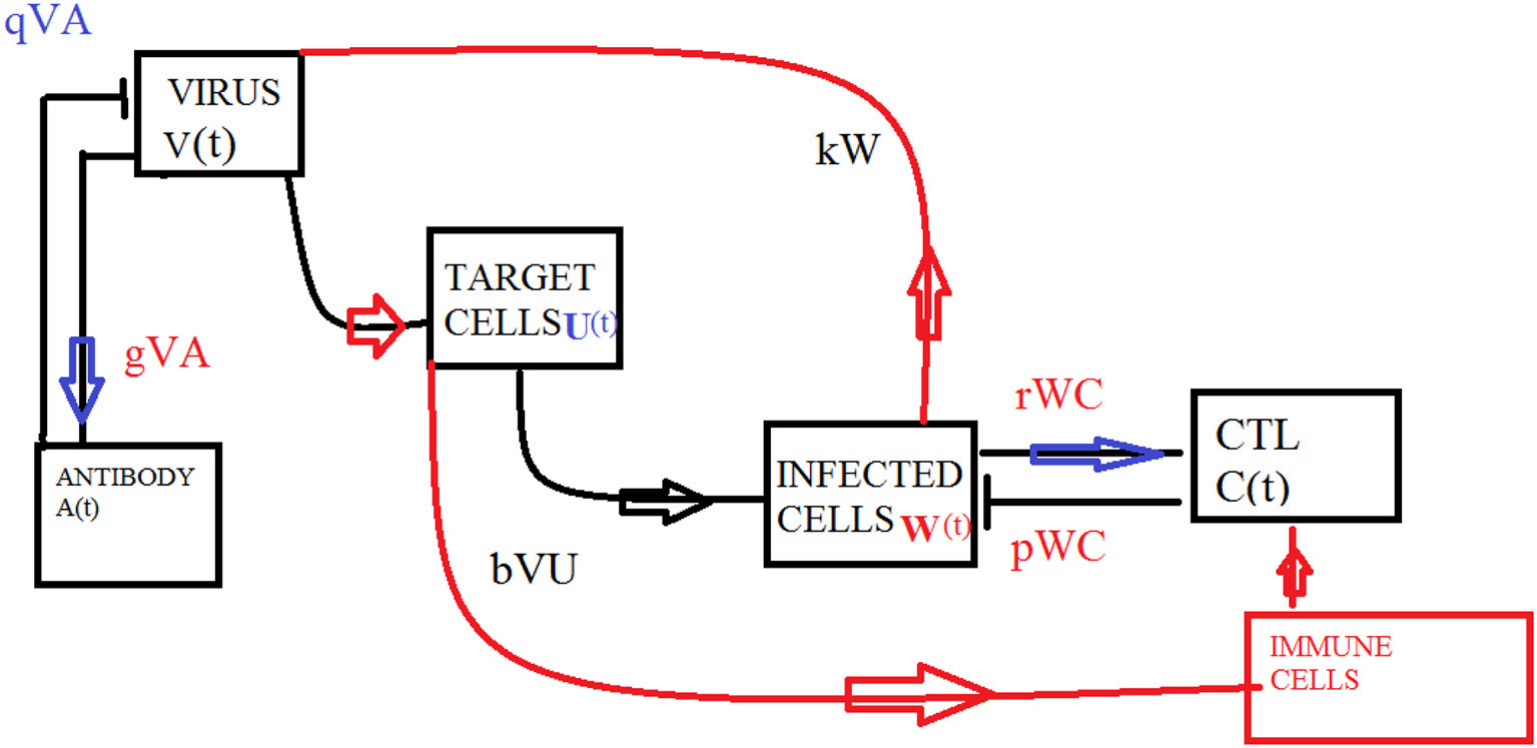
Schematic of the model.

**Figure 2:**
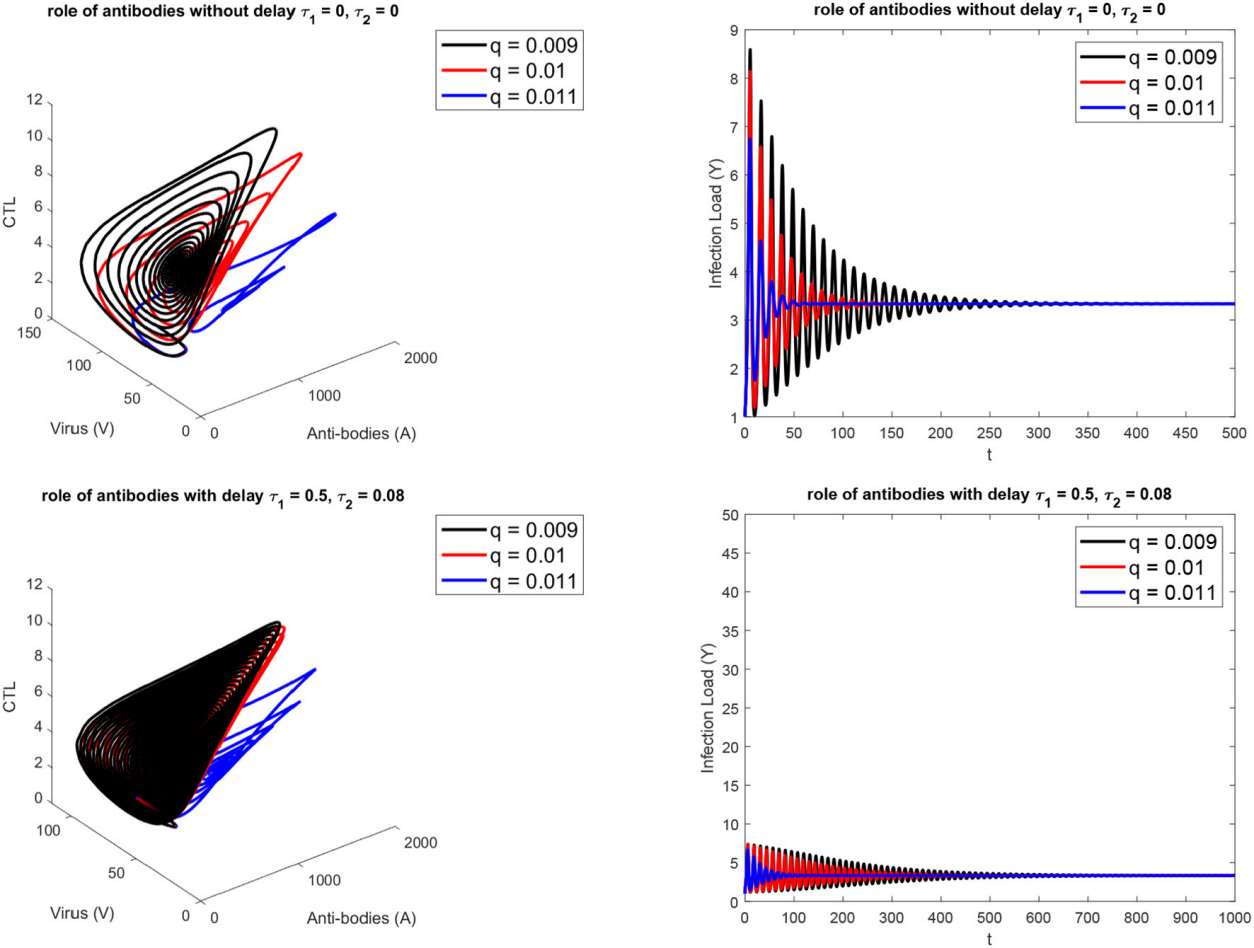
For three different values of antibody-SARS-2 interaction rates (*q*). **(Top)** without, **(Bottom)** with delay τ_1_ = 0.5, τ_2_ = 0.08.

**Figure 3:**
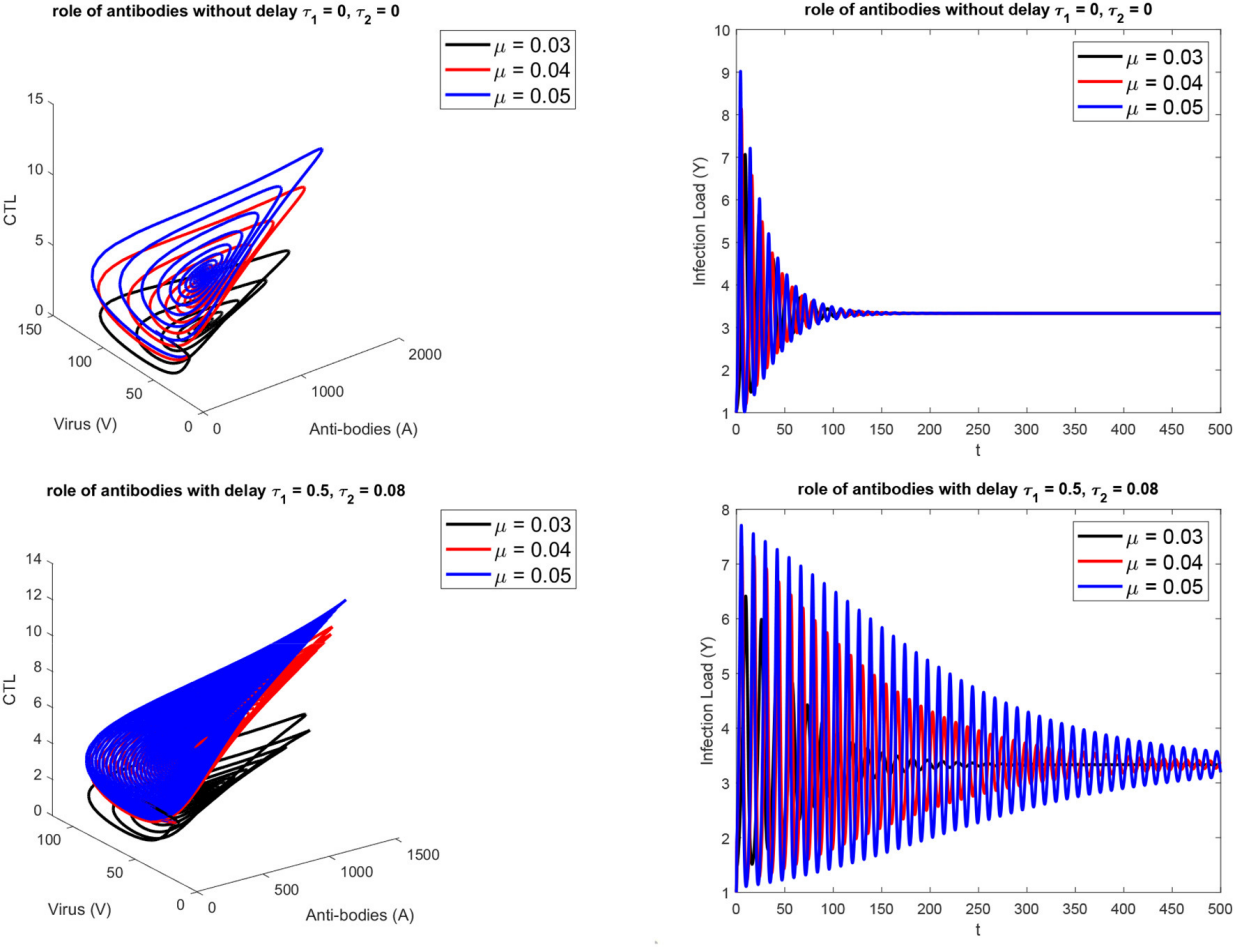
For three different values of the replication rate of virus. **(Top)** without, **(Bottom)** with delay τ_1_ = 0.5, τ_2_ = 0.08.

**Figure 4:**
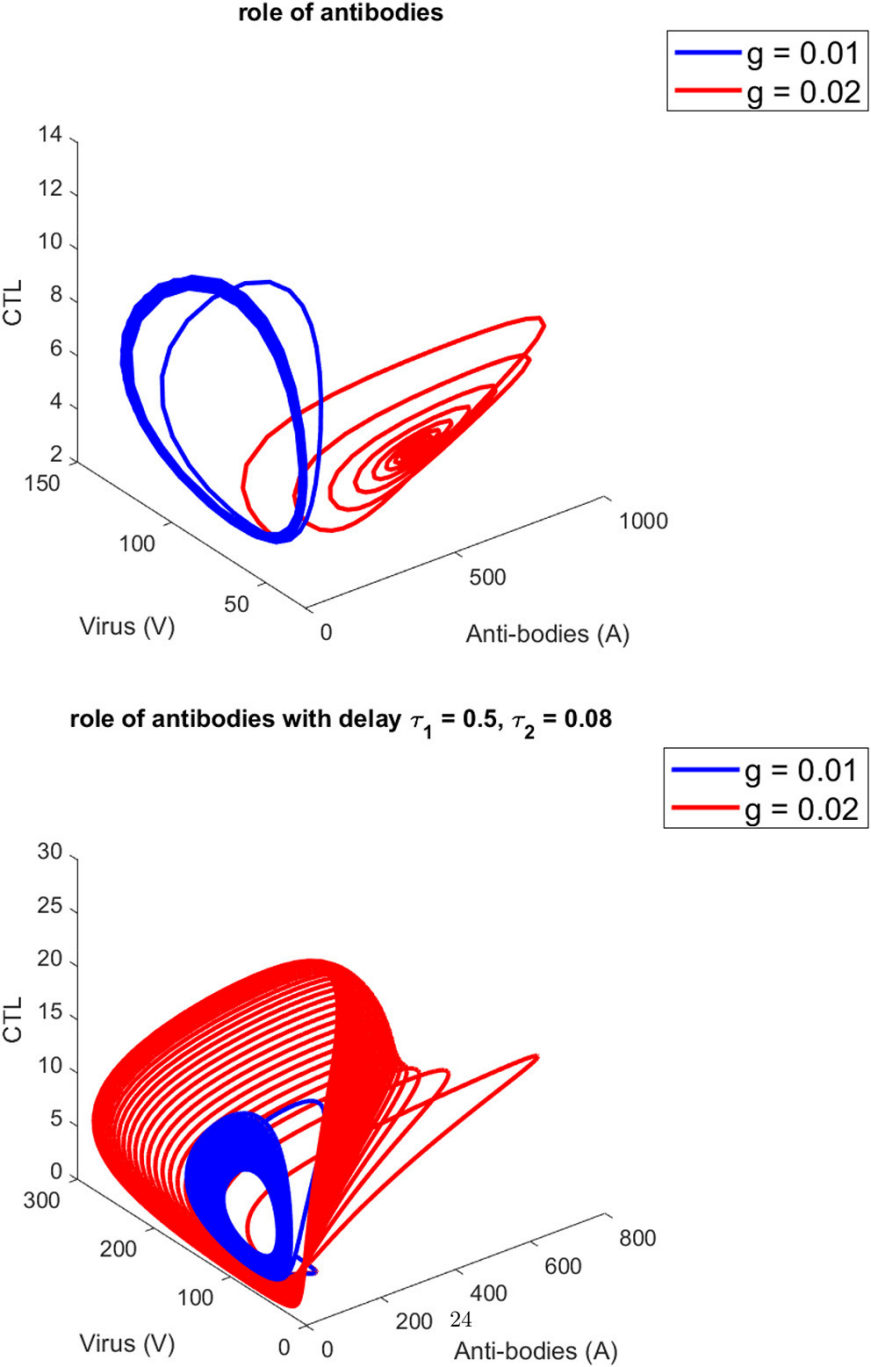
For three different values of *g*. **(Top)** without, **(Bottom)** (with) delay τ_1_ = 0.5, τ_2_ = 0.08.

Figure 1 presents the schematic to understand the interaction of key players. Figure 2 provides the phase space portraits to explore the interaction between the CTL's, the anti bodies and the virus, for three different values of q. We can see that for increasing values of q, there is reduction in the concentration of CTL, as well as the length of the cycle increases over time (top panel). On the other hand, when delay was considered, the dynamics were different. Figure 3 provides analysis relative to the replication rate of virus. Figure 4 presents the dynamics relative to the parameter g. We can see a twist in the phase space portraits when the delay was taken into account. In the supplementary figures, (Figures S1 and S2, we can see the dynamics more clearly relative to change in parametric values). We can see that the dynamics are more visible to witness the rapid action of SARS-2. Thus, a model without delay, and with η = 0 will not be able to demonstrate the dynamics well. We thus conclude that the disease transmission and the immune response depends on time delay as well as nonlinear Hill formalism.

## 4. Conclusions and Future Work

The manuscript presents a state of the art model, with delay, from one compartment to the next due to transition. In nature, there is always a delay in the onset of infections. The proposed mathematical model quantifies and analyzes this imbalance and describes the temporal trend of the phenomenon, leaving its application open to possible direct therapies in that sense.

- Mathematical analysis of the non-lytic and lytic action of the immune reaction to SARS-CoV2.
- Construction of a model that describes the balance between antibody reaction and cellular reaction mediated by CTL cells.
- Analysis of the imbalance between non-lytic and lytic action of the immune response.
- Description and quantification of the model related to the infection and functionality of CTL cells over time.
- Evidence of delay in disease transmission.

## Data Availability Statement

The original contributions generated for the study are included in the article/Supplementary Material, further inquiries can be directed to the corresponding author/s.

## Author Contributions

ZY and AS did the modeling. AN and RA did the literature review and simulations. All of the authors equally contributed to the manuscript and participated in results and discussion.

## Conflict of Interest

The authors declare that the research was conducted in the absence of any commercial or financial relationships that could be construed as a potential conflict of interest.
