# Supplementary figures and images for: Delayed Modeling Approach to Forecast the Periodic Behavior of SARS-2
Source: Front Mol Biosci. 2021 Apr 1;7:585245. doi: 10.3389/fmolb.2020.585245 (PMC8047460; doi:10.3389/fmolb.2020.585245)

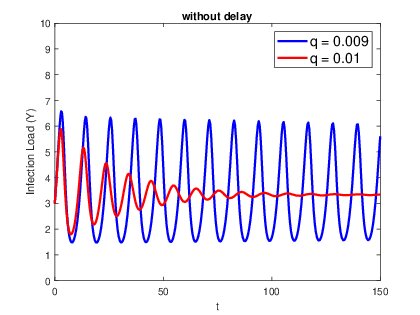

Supplement: Supplementary file 1 [file Image_1.jpg]

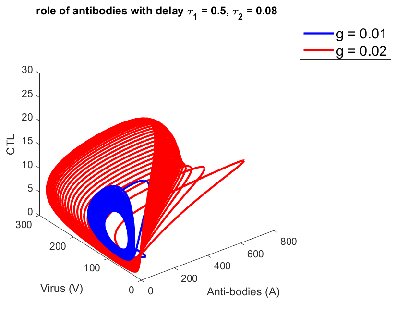

Supplement: Supplementary file 2 [file Image_2.jpg]
